# Mild Encephalitis/Encephalopathy With a Reversible Splenial Lesion (MERS) Type II in an Adult: A Case Report and Diagnostic Insight

**DOI:** 10.7759/cureus.95265

**Published:** 2025-10-23

**Authors:** Lingling Zhuang

**Affiliations:** 1 Neurology, Quangang District Hospital, Quanzhou, CHN

**Keywords:** corpus callosum, encephalopathy, mers, mild encephalitis/encephalopathy with a reversible splenial lesion, reversible splenial lesion

## Abstract

Mild encephalitis/encephalopathy with a reversible splenial lesion (MERS) is a rare clinico-radiological syndrome characterized by transient lesions in the splenium of the corpus callosum on magnetic resonance imaging (MRI). Although increasingly recognized in pediatric populations, MERS remains underdiagnosed in adults. We report a case of adult-onset MERS that presented with nonspecific neurological symptoms following a respiratory prodrome. Brain MRI demonstrated the characteristic diffusion-restricting splenial lesion, which guided the diagnostic approach. The rapid and complete resolution of both clinical symptoms and radiological abnormalities confirmed the diagnosis. This case highlights the importance of including MERS in the differential diagnosis of adult encephalopathy, particularly due to its excellent prognosis and potential to mimic more serious neurological conditions.

## Introduction

First described by Tada et al. in 2004, mild encephalitis/encephalopathy with a reversible splenial lesion (MERS) is now recognized as a distinct clinico-radiological entity [1]. Radiologically, this condition is characterized by a reversible lesion within the splenium of the corpus callosum (SCC), which typically presents as a focal area of high signal intensity on T2-weighted, fluid-attenuated inversion recovery (FLAIR), and notably on diffusion-weighted imaging (DWI) sequences, with correspondingly reduced apparent diffusion coefficient (ADC) values [1,2]. Although more frequently reported in children, MERS is considered to be underrecognized in adult populations, posing diagnostic challenges [3]. Patients typically present with mild neurological symptoms, such as altered mental status, headache, or seizures, often following a prodromal infection. The distinctive radiological findings are paramount to accurate diagnosis, enabling the distinction of MERS from other neurological conditions with similar presentations but less favorable outcomes. This report presents an instructive adult case of MERS to highlight its characteristic imaging features and emphasize diagnostic considerations in clinical practice.

## Case presentation

A 28-year-old man presented to the respiratory department with a five-day history of a prodromal respiratory illness, characterized by productive cough with yellowish-white sputum, recurrent fever (peak temperature 38.2°C), nasal congestion, nausea, and vomiting. His symptoms were unresponsive to self-administered antipyretics and cough suppressants. On the day of admission, the patient developed acute neurological deterioration in the evening. The new symptoms included delayed responsiveness, slurred speech, and trismus (i.e., difficulty opening the mouth). He specifically denied headaches, loss of consciousness, or convulsive activity.

On examination, the patient was febrile (38.9°C) and was drowsy with significantly impaired verbal responses. Cranial nerve assessment revealed right-sided orofacial weakness and mild restriction in mouth opening and tongue protrusion. Motor and sensory examinations revealed decreased superficial sensation on the right hemiface and upper limb; limb power and tone were normal. Plantar responses were flexor bilaterally.

Initial laboratory investigations revealed significant hyponatremia (120.0 mmol/L) and hypokalemia, as well as elevated creatine kinase (1,744 U/L) and C-reactive protein (27.99 mg/L). Chest CT demonstrated bilateral pulmonary infiltrates consistent with pneumonia and small pleural effusions. Comprehensive infectious disease testing, including assays for influenza, SARS-CoV-2, and other respiratory pathogens, yielded negative results. It is important to note that the patient declined further investigations, including electroencephalography (EEG) and lumbar puncture for cerebrospinal fluid analysis or autoimmune testing. Therefore, the diagnosis relied predominantly on clinical and radiological findings.

Neuroimaging findings

**Figure 1:**
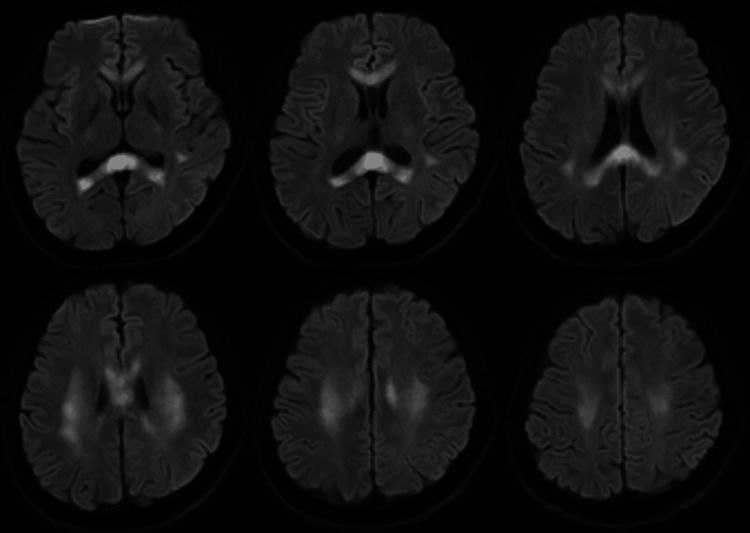
Initial brain MRI showing high signal intensity on DWI sequences in the splenium of the corpus callosum and bilateral frontal-parietal white matter. MRI: magnetic resonance imaging; DWI: diffusion-weighted imaging

At the time of admission, brain magnetic resonance imaging (MRI) was performed. The images demonstrated conspicuous high signal intensity on DWI sequences localized to the SCC and extending to the bilateral frontal-parietal white matter, findings consistent with MERS type II (Figure 1).

Following the MRI findings, a diagnosis of MERS was strongly suspected. The patient was started on supportive care, including anti-infectives for the concomitant pneumonia and measures to correct hyponatremia.

**Figure 2:**
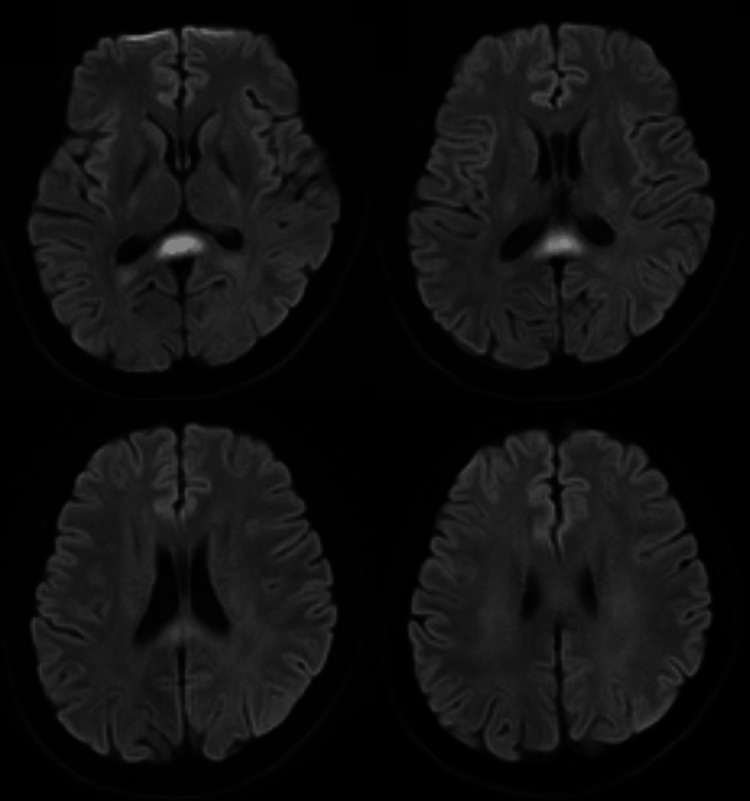
Follow-up MRI performed five days after the initial scan showing near-complete resolution of the previously noted DWI hyperintensities. MRI: magnetic resonance imaging; DWI: diffusion-weighted imaging

A mere five days after the initial scan, a follow-up MRI was performed. This study revealed a dramatic and nearly complete resolution of the previously noted DWI hyperintensities (Figure 2). This rapid radiological improvement was mirrored by the patient's clinical condition; his neurological symptoms resolved completely within one week of supportive management, thereby solidifying the diagnosis of MERS.

Clinical Pearl

The combination of mild encephalopathic symptoms following a prodromal illness and the characteristic reversible splenial lesion on MRI should lead to the consideration of MERS in adult patients. The rapid resolution of both clinical and radiological findings is diagnostically reassuring.

## Discussion

The present case exemplifies the classic presentation and clinical course of MERS in an adult patient. The diagnosis fundamentally relies on the recognition of the characteristic neuroimaging pattern. The transient nature of the lesion, which typically resolves within days to weeks without sequelae, is pathognomonic [1,4]. Although the most common MRI presentation is an isolated ovoid lesion confined to the SCC (MERS type I), extension to adjacent white matter regions, as observed in this case, defines MERS type II [5]. The striking DWI hyperintensity and corresponding ADC hypointensity are thought to reflect intramyelinic edema due to fluid accumulation between the myelin layers, potentially influenced by inflammatory cytokines or associated metabolic disturbances, as exemplified by the prominent hyponatremia in this case [6,7]. MERS type II is rare in adults, with limited incidence data; however, it is increasingly recognized in the literature, particularly in association with infections and metabolic imbalances [3,5].

The principal clinical challenge in MERS lies in its timely recognition and appropriate differentiation from other neurological disorders that can affect the corpus callosum. During the diagnostic process, the following alternatives were carefully considered.

Acute disseminated encephalomyelitis

This disease typically presents with multifocal, asymmetric lesions that may demonstrate contrast enhancement and resolve more gradually over weeks to months [8].

Marchiafava-Bignami disease

This disease is strongly associated with chronic alcoholism and characteristically involves the entire body of the corpus callosum rather than showing splenium-predominant involvement [9].

Ischemic stroke

It usually conforms to a specific vascular territory and results in irreversible damage, contrasting sharply with the transient nature of MERS lesions [10].

Multiple sclerosis

Multiple sclerosis (MS) demonstrates a relapsing-remitting clinical course with characteristic periventricular and callososeptal lesions that rarely resolve completely [11].

The rapid clinical improvement, coupled with complete radiological resolution in the patient, effectively excluded these alternative diagnoses. Management of MERS is primarily supportive, focusing on treating the underlying trigger (often an infection) and correcting associated metabolic derangements [3,6]. The role of immunomodulatory therapies, such as corticosteroids or intravenous immunoglobulins, remains debated in the literature, particularly since most patients experience complete recovery without such interventions, as demonstrated in the present case [2]. The highly favorable prognosis underscores the importance of accurate diagnosis, which alleviates patient and family anxiety and prevents unnecessary aggressive treatments or prolonged hospital stays [1,3].

## Conclusions

MERS is a clinico-radiological syndrome characterized by transient lesions in the SCC. MERS should be a key consideration in adults presenting with encephalopathy following a prodromal illness. This case demonstrates that prompt neuroimaging, specifically DWI, is essential for identifying the characteristic splenial lesion. Recognizing this imaging signature allows clinicians to confidently diagnose MERS, differentiate it from serious alternatives, and reassure patients regarding the expected excellent recovery with supportive management. Increased recognition of this entity is essential for optimal management.
